# A multicenter randomized open-label phase 2 study investigating optimal antiemetic therapy for patients with advanced/recurrent gastric cancer treated with trastuzumab deruxtecan: the EN-hance study

**DOI:** 10.1007/s10147-025-02748-8

**Published:** 2025-04-28

**Authors:** Toru Aoyama, Akira Ooki, Koji Oba, Kazuhiro Nishikawa, Ryohei Kawabata, Michitaka Honda, Hiromichi Maeda, Mitsuro Kanda, Keiji Sugiyama, Akitaka Makiyama, Kenki Segami, Masazumi Takahashi, Yoshiaki Shindo, Tsutomu Namikawa, Takashi Oshima, Aya Katayama, Kazuhito Shiosakai, Junichi Sakamoto

**Affiliations:** 1 Department of Surgery, Yokohama City University Hospital, Yokohama, Japan; 2 The Cancer Institute Hospital of Japanese Foundation for Cancer Research, Koto, Japan; 3 The University of Tokyo Graduate School of Medicine, Bunkyō, Japan; 4 Osaka Police Hospital, Osaka, Japan; 5 Sakai City Medical Center, Sakai, Japan; 6 Fukushima Medical University, Fukushima, Japan; 7 Kochi Medical School Hospital, Nankoku, Japan; 8 Nagoya University Graduate School of Medicine, Nagoya, Japan; 9 NHO Nagoya Medical Center, Nagoya, Japan; 10 Gifu University Hospital, Gifu, Japan; 11 Saiseikai Yokohama-Shi Nanbu Hospital, Yokohama, Japan; 12 Yokohama Municipal Citizen’s Hospital, Yokohama, Japan; 13 Nakadori General Hospital, Akita, Japan; 14 Kanagawa Cancer Center Hospital, Yokohama, Japan; 15 Daiichi Sankyo Co., Ltd., Tokyo, Japan; 16 Tokai Central Hospital, Kakamigahara, Japan; 17 Present Address: Department of Gastric Surgery, Tokyo Metropolitan Cancer and Infectious Diseases Center Komagome Hospital, Tokyo, Japan

**Keywords:** Phase 2 study, Antiemetic therapy, Gastric cancer, Trastuzumab deruxtecan

## Abstract

**Background:**

Trastuzumab deruxtecan (T-DXd) has been approved for the treatment of human epidermal growth factor receptor-2 (HER2)-positive gastric cancer and other indications in several countries and is considered moderately or highly emetogenic. The management of nausea and vomiting associated with T-DXd treatment has not been fully evaluated and the effectiveness of conventional prophylaxis remains unknown.

**Methods:**

This open-label, randomized, multicenter, phase 2 study aimed to investigate the optimal antiemetic therapy for Japanese patients with gastric cancer undergoing T-DXd treatment. Patients were randomized to a doublet regimen group (dexamethasone and palonosetron) or triplet regimen group (aprepitant, dexamethasone, and palonosetron) at a ratio of one to one, stratified by sex, gastrectomy status, and study institution. Both antiemetic treatments were administered from day 1 before T-DXd administration, and emetic events and nausea were observed for 21 days. The primary endpoint was the antiemetic complete response (CR) rate to assess control for emetic events based on voluntary patient-reported outcomes (PROs) during cycle 1 (1–21 days).

**Results:**

Of the 60 enrolled patients, 58 were eligible for inclusion in this analysis (29 patients in each regimen group). The overall CR rates for the doublet and triplet regimens were 41.4% (12/29 patients) and 37.9% (11/29 patients), respectively, and neither regimen met the pre-specified threshold (> 18/29 patients). The CR rate in the acute phase (0–24 h) was 86.2% (25/29 patients) for both regimens, and the CR rates in the delayed phase (2–21 days) were 41.4% (12/29 patients) and 37.9% (11/29 patients) for the doublet and triplet regimens, respectively.

**Conclusions:**

Given that the primary endpoint was not met, further research is needed to better characterize nausea and vomiting with T-DXd to tailor an anti-emetic regimen that suits the needs of the patients.

## Introduction

Gastric cancer (GC) is one of the most common cancers worldwide, causing more than 700,000 deaths annually [1]. Systemic chemotherapy is the standard treatment for patients with advanced unresectable GC [2, 3]. Despite improvements in treatments for advanced GC, the prognosis of advanced GC is still poor, with a median survival of 12–16 months [4–6].

Recently, personalized treatments have been introduced for advanced GC. In 2010, the ToGA trial demonstrated that trastuzumab, an anti-human epidermal growth factor receptor-2 (HER2) monoclonal antibody, with systemic chemotherapy improved the survival of patients with HER2-positive advanced GC [7]. According to the ToGA trial, the HER2 status of advanced GC significantly changed the treatment strategy [8]. In 2020, the DESTINY-Gastric01 trial showed that trastuzumab deruxtecan (T-DXd), an antibody–drug conjugate consisting of a humanized monoclonal anti-HER2 antibody bound to a cytotoxic topoisomerase I inhibitor (drug payload) by means of a cleavable tetra peptide-based linker, significantly improved the overall survival of HER2-positive advanced GC [9]. Currently, T-DXd is a key drug widely used to treat HER2-positive advanced GC [10]. However, effective management of treatment-emergent vomiting and nausea is needed in order for more patients to benefit from longer T-DXd treatment [11].

In the DESTINY-Gastric01 trial, the incidence of any-grade vomiting was 26% and nausea was 63% [9]. Recently, the National Comprehensive Cancer Network (NCCN) guidelines in oncology for antiemesis was updated to list T-DXd as highly emetogenic [12].

However, the risk of emesis in patients receiving T-DXd treatment has not been fully evaluated, and the effectiveness of conventional prophylaxis remains unknown, lending the opportunity to further characterize these events. Thus, it is necessary to clarify the frequency and duration of nausea and vomiting caused even with prophylactic administration of each antiemetic medication after T-DXd dosing, which will lead to optimal prophylactic regimen for emesis. This is likely to lead to improved treatment outcomes as well as extended survival due to the improved continuity and length of the T-DXd treatment. It is also expected to improve the quality of life.

Therefore, we conducted an open-label, randomized, multicenter, phase 2 study to investigate specific antiemetic regimens for patients with unresectable and/or recurrent GC undergoing T-DXd treatment.

## Patients and methods

### Patients

Eligible patients were older than 20 years of age with an Eastern Cooperative Oncology Group (ECOG) performance status (PS) of 0–2, and confirmed HER2-positive (Immunohistochemistry [IHC] 3 + or IHC 2 and in situ hybridization [ISH]-positive) gastric or gastroesophageal junction cancer. Patients scheduled to receive T-DXd as a third-or-later-line treatment were eligible. Patients were required to have adequate organ functions, including aspartate aminotransferase ≤ 126 U/L, alanine aminotransferase ≤ 126 U/L, total bilirubin ≤ 2.5 mg/dL, and creatinine clearance ≥ 30 mL/min. Patients with complications, a history of interstitial lung disease, or symptomatic brain metastases were excluded from the analysis. Patients with a history of T-DXd therapy, Common Terminology Criteria for Adverse Events (CTCAE) grade ≥ 2 nausea or vomiting at enrollment, and a history of hypersensitivity to neurokinin 1 (NK1) receptor antagonist, 5-HT3 receptor antagonist, dexamethasone (DEX), trastuzumab, or excipients of T-DXd were considered ineligible.

### Study design

This open-label, randomized, multicenter phase 2 study was conducted at 29 institutions in Japan. The study was conducted in accordance with the Declaration of Helsinki and the Clinical Trials Act and was approved by the CRB of Yokohama City University. Additionally, written informed consent was obtained from all patients. This study was registered in the Japan Registry of Clinical Trials (identifier: jRCTs031200336).

The primary endpoint was the complete response (CR) rate after T-DXd administration up to day 21 (during the overall period). CR was defined as the absence of emetic events or rescue medication during the study period. The CR rate was defined as the proportion of complete responders. Among the efficacy measures representing the control status of emetic events, the CR rate during the overall period has been used as the primary endpoint in many clinical studies evaluating the usefulness of antiemetic agents. Therefore, the overall CR rate was adopted as the primary endpoint in this study.

The secondary endpoints were CR in the acute phase (0–24 h) and delayed phase (days 2–21), complete control (no emetic events, no rescue medication, and no significant nausea), and total control (no emetic events, no rescue medication, and no nausea).

Overall survival (OS; defined as the time from the date of randomization [the date of registration for Exploratory Cohorts] to the date of death due to any cause), time to treatment failure (TTF; time to the first emetic episode or use of rescue medication), and safety were evaluated.

The patients used a diary to record emetic events, the rescue medications that they used for emetic events or nausea, and their response to the rescue medication from days 1 to 21. Emetic events were defined as vomiting and retching, and the number of events and onset time of the first event each day until day 21 were recorded. Emetic events occurring within an interval of less than 1 min were counted as a single episode. Nausea was recorded on a four-item Likert scale (no nausea, mild, moderate, or severe) each day until day 21. Nausea was recorded as the most severe intensity, according to an 11-point numerical rating scale (NRS), each day until day 21.

Adverse events were assessed for frequency and grade over the 21 day period according to CTCAE version 5.0 [13]. OS was assessed one year after the date of enrollment of the last patient.

### Randomization

Eligible patients were randomized (1:1) to receive either dexamethasone and palonosetron (doublet regimen) or dexamethasone, palonosetron, and aprepitant or fosaprepitant (triplet regimen). The allocation of patients was centrally performed using non-deterministic minimization methods with adjustment factors including sex, gastrectomy status, and study institution. The data center prepared the randomization list and managed the patients’ allocation information.

### Treatment

Antiemetic regimens were selected according to Japanese guidelines for the proper use of antiemetics [14]. Patients receiving the doublet regimen were administered DEX (9.9 mg intravenous infusion 30 min prior to the start of T-DXd on day 1 and 8 mg oral administration on days 2 and 3) and palonosetron (0.75 mg intravenous infusion 30 min prior to the start of T-DXd). The administration of DEX on day 4 was allowed depending on the patient’s condition.

Patients receiving the triplet regimen received DEX (9.9 mg intravenous infusion 30 min prior to the start of T-DXd on day 1 and 8 mg orally administered on days 2, 3, and 4) and palonosetron (0.75 mg intravenous infusion 30 min prior to the start of T-DXd on day 1). An NK1 receptor antagonist (125 mg of aprepitant orally administered 60 min prior to the start of T-DXd on day 1 and 80 mg orally administered on days 2 and 3, or 150 mg fosaprepitant intravenous infusion 60 min on day 1 prior to T-DXd) was also administered. Patients were allowed to take rescue medications for emetic events, except for NK1 receptor antagonists, during cycle 1. The administration of DEX on day 5 was allowed depending on the patient’s condition. All patients received T-DXd (6.4 mg/kg) on day 1 of the 21 day cycle.

### Statistical analyses

This study adopted the randomized 2-stage phase 2 study design proposed by Hou et al. [15]. In previous non-inferiority trials, a non-inferiority margin of 15% was adopted for the overall CR rate [15, 16]. This margin was also set as the clinically important difference in this study. The safety analysis in a phase 1 study of T-DXd for GC showed that the incidence rate of vomiting was 52.5% in patients with no scheduled antiemetic treatment. Based on these reports, we set a threshold CR value of 45% in the present study [16]. With the threshold CR rate set at 45%, the expected CR rate at 70% [9], and values of 1-sided alpha 1 = 0.05, beta 1 = 0.2, 1-sided alpha 2 = 0.05, and beta 2 = 0.3, according to Hou et al.’s design parameter, the required sample size was calculated as 29 patients per arm, and 58 patients in total.

For the primary analysis, the evaluation of a promising antiemetic regimen was performed as follows: (1) if the null hypothesis that the number of CR patients was < 18 of the 29 patients could not be rejected in both regimens, the study determined that both regimens were not promising; (2) if ≥ 18 CR patients out of the 29 patients were observed in only one arm, the antiemetic regimen in that arm was deemed promising; and (3) if the null hypothesis was rejected in both regimens, and the difference in the number of CR patients between the two regimens was < 3 patients, then both regimens were considered promising. If the difference in the number of CR patients between regimens was 3, antiemetic treatment with a higher CR rate was considered promising. We also calculated the overall CR rates in each arm, along with their 90% confidence intervals, based on the Agresti-Coull method. In addition, the difference in the overall CR rates between the two arms was calculated, and the 90% confidence interval of the Newcombe score was computed. In cases where the presence or absence of emetic events could not be determined due to missing data, it was treated as if CR was not achieved and the case was not excluded from the denominator.

The CR rate in the acute and delayed phases, complete control rate, and total control rate for each arm were calculated using the same methods as in the primary analysis. TTF curves and their median values were estimated using the Kaplan–Meier method. The log-rank test was used for the statistical analysis. Adverse events were compared using the Fisher’s exact test. We investigated the clinicopathological factors associated with nausea and vomiting. For each factor, we employed modified least squares regression to calculate the difference in incidence proportions and corresponding 95% confidence intervals [17]. A multivariable analysis was then conducted based on the variables selected using a backward elimination method (selection criterion: *p* < 0.20). All statistical analyses were performed using SAS software version 9.4.

## Results

### Patients

**Fig. 1:**
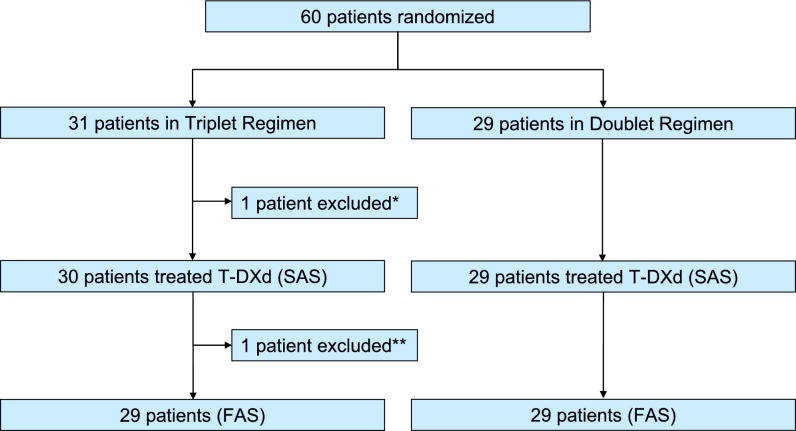
Consort diagram of the present study. ^*^One patient was excluded due to inability to receive treatment with T-DXd for disease progression, ^**^ One patient was excluded due to Informed consent violation. SAS: safety analysis set, FAS: full analysis set

Sixty patients were enrolled in this study between February 2021 and October 2022. These patients were randomized, and the safety analysis was ultimately conducted in 59 patients after excluding 1 patient in the triplet regimen group because they did not receive T-DXd for disease progression. Furthermore, one patient was excluded due to non-compliance with obtaining informed consent, 58 patients (29 patients in each regimen) were ultimately included in the efficacy analysis of the primary and secondary endpoints (Fig. 1).

Across both regimens, the median age was 72 years, and the PS was 0, 1, and 2 in 31 (53.4%), 25 (43.1%), and 2 (3.4%) patients, respectively. Forty patients (69.0%) had received 2 previous systemic therapies for advanced or metastatic disease, and 18 (31.0%) had received ≥ 3 previous systemic therapies. In addition, 44 patients (75.9%) had previously received a platinum-containing regimen. The relative dose intensity of T-DXd was also the same between the two groups. The demographic data and patient characteristics are listed in Table 1. Gastrectomy status, age, BMI, drinking history within 30 days, previous use of immune check point inhibitor, and laboratory data including serum albumin level was almost similar between two groups.

**Table 1:** Clinical characteristics of study patients

Patient characteristic	Triplet regimen (*N* = 29)	Doublet regimen (*N* = 29)	Total (*N* = 58)
Age			
Median (range)	72.0 (53–83)	72.0 (41–82)	72.0 (41–83)
Sex, *n* (%)			
Female	7 (24.1)	6 (20.7)	13 (22.4)
Male	22 (75.9)	23 (79.3)	45 (77.6)
Body mass index			
Median (range)	19.50 (13.5–27.3)	21.00 (16.7–27.8)	20.75(13.5–27.8)
ECOG performance status, *n* (%)			
0	14 (48.3)	17 (58.6)	31 (53.4)
1	14 (48.3)	11 (37.9)	25 (43.1)
2	1 (3.4)	1 (3.4)	2 (3.4)
HER2 status, *n* (%)			
IHC3 +	21 (72.4)	20 (69.0)	41 (70.7)
IHC2 + and ISH positive	8 (27.6)	9 (31.0)	17 (29.3)
Histological type (%)			
Intestinal	24 (82.8)	26 (89.7)	50 (86.2)
Diffuse	4 (13.8)	2 (6.9)	6 (10.3)
Other	1 (3.4)	1 (3.4)	2 (3.4)
Previous systemic therapies for advanced or metastatic disease (%)			
2 line	21 (72.4)	19 (65.5)	40 (69.0)
≥ 3 line	8 (27.6)	10 (34.5)	18 (31.0)
Gastrectomy (%)			
Yes	12 (41.4)	14 (48.3)	26 (44.8)
No	17 (58.6)	15 (51.7)	32 (55.2)
Previous platinum regimen (%)			
Yes	22 (75.9)	22 (75.9)	44 (75.9)
No	7 (24.1)	7 (24.1)	14 (24.1)
Previous immune check point inhibitor (%)			
Yes	10 (34.5)	8 (27.6)	18 (31.0)
No	19 (65.5)	21 (72.4)	40 (69.0)

*ECOG* Eastern Cooperative Oncology Group, *HER*2 human epidermal growth factor receptor 2

### Antiemetic efficacy

The treatment compliance rates of the scheduled antiemetic treatment were 96.6% (28/29) and 100% for the doublet and triplet regimens, respectively. One patient in the doublet regimen group did not receive DEX on days two or three.

The antiemetic CR rate during the overall period (the primary endpoint) was 41.4% (12/29, 90% CI 27.7–56.5%) in the doublet regimen group and 37.9% (11/29, 90% CI 24.7–53.2%) in the triplet regimen group. Three patients in the doublet regimen group and five in the triplet regimen group were considered as having no CR because of incomplete patient diaries.

The null hypothesis that the number of CR was < 18 out of 29 patients could not be rejected in either regimen, so the trial determined that neither regimen was promising. The CR rate during the acute phase was 86.2% (25/29, 90% CI 72.2–94.1%) in both regimens, and the rates during the delayed phase were the same as those during the overall period. The CR rates during the first five days were 51.7% (15/29, 90% CI 37.0–66.2%) in the doublet regimen group and 55.2% (16/29, 90% CI 40.2–69.3%) in the triplet regimen group. The complete control and total control rates during the overall period, acute and delayed phases, and the first 5 days are shown in Table 2.

**Table 2:** Proportion of patients achieving CR during each phase

	Triplet regimen (*N* = 29)	Doublet regimen (*N* = 29)
Complete response (CR), *n* (%; 90%CI)		
Overall period (day 1–day 21)	11 (37.9; 24.7, 53.2)	12 (41.4; 27.7, 56.5)
Acute phase (0–24 h)	25 (86.2; 72.2, 94.1)	25 (86.2; 72.2, 94.1)
Delayed phase (day 2–day 21)	11 (37.9; 24.7, 53.2)	12 (41.4; 27.7, 56.5)
First 5 days (day 1–day 5)*	16 (55.2; 40.2, 69.3)	15 (51.7; 37.0, 66.2)
Complete control (CC), *n* (%; 90%CI)		
Overall period (day 1–day 21)	9 (31.0; 19.0, 46.4)	11 (37.9; 24.7, 53.2)
Acute phase (0–24 h)	24 (82.8; 68.3, 91.7)	25 (86.2; 72.2, 94.1)
Delayed phase (day 2–day 21)	10 (34.5; 21.8, 49.8)	11 (37.9; 24.7, 53.2)
First 5 days (day 1–day 5)*	16 (55.2; 40.2, 69.3)	15 (51.7; 37.0, 66.2)
Total control (TC), *n* (%; 90%CI)		
Overall period (day 1–day 21)	5 (17.2; 8.3, 31.7)	10 (34.5; 21.8, 49.8)
Acute phase (0–24 h)	23 (79.3; 64.5, 89.1)	23 (79.3; 64.5, 89.1)
Delayed phase (day 2–day 21)	6 (20.7; 10.9, 35.5)	10 (34.5; 21.8, 49.8)
First 5 days (day 1–day 5)*	11 (37.9; 24.7, 53.2)	14 (48.3; 33.8, 63.0)

*First 5 days is exploratory analysis

**Fig. 2:**
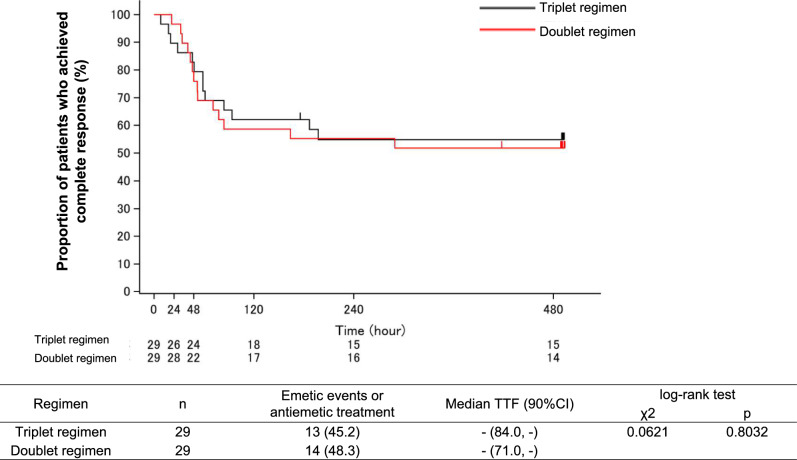
Time to treatment failure

The Kaplan–Meier analysis of TTF is shown in Fig. 2. There was no statistically significant difference between the doublet and triplet regimens (*p* = 0.803).

### The onset and duration of emetic events and nausea

**Fig. 3:**
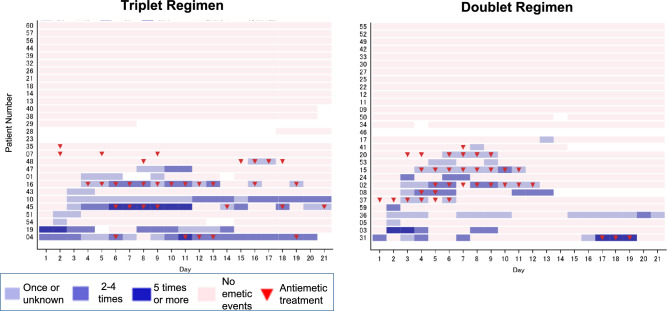

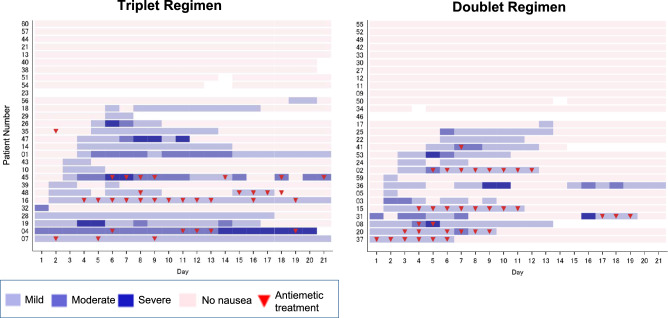
**A** The onset and duration of emetic events for each patient. **B** The onset and duration of nausea for each patient

The onset and duration of emetic events and the number of episodes per day for each patient are shown in Fig. 3A. In patients who experienced emetic events, the median time to the onset of the first emetic event was 3.0 (range, 2–13) days in the doublet regimen group and 3.0 (range, 1–16) days in the triplet regimen group. The median time to the onset of the first nausea episode was 2.0 (1–13) days in the doublet regimen group and 3.0 (range, 1–19) days in the triplet regimen group. The onset and duration of nausea, as well as the daily severity for each patient are shown in Fig. 3B. In patients who experienced emetic events, the median duration of such events was 3.5 (range, 1–14) days in the doublet regimen group and 4.0 (1–19) days in the triplet regimen group. The median duration of nausea was 8.0 (1–14) days in the doublet regimen group and 10.0 (range, 1–21) days in the triplet regimen group.

Based on the Likert scale assessment, 57.1% of the patients in the doublet regimen group and 67.9% of the patients in the triplet regimen group experienced nausea (mild, moderate, severe: doublet regimen, 25.0%, 17.9%, 14.3%, respectively; triplet regimen, 42.9%, 7.1%, 17.9%). The percentage of patients who experienced nausea during the acute phase was 14.3% (mild, moderate, and severe: 10.7%, 3.6%, and 0%, respectively) in the doublet regimen group and 17.9% (mild, moderate, and severe: 10.7%, 7.1%, and 0%, respectively) in the triplet regimen group.

### Safety

Adverse events of any grade were experienced by 15 patients (51.7%) in the doublet regimen group and by 23 patients (76.7%) in the triplet regimen group. Grade ≥ 3 adverse events were experienced by three patients (10.3%) in the doublet regimen group and 14 patients (46.7%) in the triplet regimen group. A summary of adverse events is presented in Table 3.

**Table 3:** Adverse events occurring in 5% or more*

Adverse Event	Triplet regimen (*N* = 30)		Doublet regimen (*N* = 29)		All (*N* = 59)	
	Any grade (%)	Grade 3/4 (%)	Any grade (%)	Grade 3/4 (%)	Any grade (%)	Grade 3/4 (%)
Hematological						
Leukopenia	2 (6.7)	1 (3.3)	2 (6.9)	0 (0.0)	4 (6.8)	1 (1.7)
Neutropenia	7 (23.3)	6 (20.0)	3 (10.3)	1 (3.4)	10 (16.9)	7 (11.9)
Anemia	4 (13.3)	2 (6.7)	3 (10.3)	1 (3.4)	7 (11.9)	3 (5.1)
Thrombocytopenia	5 (16.7)	1 (3.3)	1 (3.4)	0 (0.0)	6 (10.2)	1 (1.7)
Febrile neutropenia	3 (10.0)	3 (10.0)	2 (6.9)	1 (3.4)	5 (8.5)	4 (6.8)
Non hematological						
Anorexia	8 (26.7)	4 (13.3)	5 (17.2)	0 (0.0)	13 (22.0)	4 (6.8)
Malaise	7 (23.3)	0 (0.0)	8 (27.6)	0 (0.0)	15 (25.4)	0 (0.0)
Fatigue	4 (13.3)	1 (3.3)	2 (6.9)	0 (0.0)	6 (10.2)	1 (1.7)
Aspartate aminotransferase increased	2 (6.7)	0 (0.0)	0 (0.0)	0 (0.0)	2 (3.4)	0 (0.0)
Peripheral sensory neuropathy	2 (6.7)	0 (0.0)	1 (3.4)	0 (0.0)	3 (5.1)	0 (0.0)
Fever	2 (6.7)	0 (0.0)	0 (0.0)	0 (0.0)	2 (3.4)	0 (0.0)

*At least 5% in either regimen or total

Common Terminology Criteria for Adverse Events (CTCAE) Version5.0

### Risk factor analysis for emetic events

Table 4 shows the risk factor analysis for vomiting. In the multivariable analysis, pretreatment body mass index was risk factors for vomiting. In addition, Table 5 shows the risk factor analysis for nausea. In the multivariable analysis, no statistically significant risk factor was found for nausea.

**Table 4:** Uni and multivariable risk factor analysis of clinicopathological factors for vomiting

Variables	Univariable analysis		Multivariable analysis	
	Risk difference (95%CI)	*p* Value	Risk difference (95%CI)	*p* Value
Age (year)				
> 65 vs < 65	0.082 (− 0.269, 0.436)	0.6382		
Gender				
Female vs Male	0.039 (− 0.297, 0.375)	0.8154		
Pretreatment body mass index				
> 20.75 vs < 20.75	− 0.310 (− 0.567, − 0.054)	0.0185	− 0.322 (− 0.580, − 0.063)	0.0157
ECOG performance status				
1, 2 vs 0	0.025 (− 0.246, 0.296)	0.8533		
Use of immune check point inhibitor				
Yes vs No	0.019 (− 0.276, 0.315)	0.8957		
Gastrectomy				
Yes vs No	0.195 (− 0.073, 0.462)	0.1506	0.239 (− 0.010, 0.488)	0.0600
Antiemetic Therapy				
Two-drug vs Three-drug	0.103 (− 0.165, 0.372)	0.4432		
Pretreatment serum albumin (g/dl)				
> 3.5 vs < 3.5	− 0.126 (− 0.395, 0.144)	0.3539	− 0.221 (− 0.459, 0.018)	0.0689
Number of previous treatments				
3 vs 2	0.019 (− 0.276, 0.315)	0.8957		
Previous platinum regimen				
Yes vs No	− 0.185 (− 0.508, 0.138)	0.2553		
Previous smoking habit				
Yes vs No	− 0.100 (− 0.396, 0.196)	0.5018		
Drinking history within 30 days				
Yes vs No	0.091 (− 0.235, 0.417)	0.5785	0.276 (− 0.016, 0.567)	0.0631

**Table 5:** Uni and multivariable risk factor analysis of clinicopathological factors for nausea

Variables	Univariable analysis		Multivariable analysis	
	Risk difference (95% CI)	*p* Value	Risk difference (95% CI)	*p* Value
Age (year)				
> 65 vs < 65	− 0.153 (− 0.484, 0.178)	0.3586		
Gender				
Female vs Male	− 0.183 (− 0.517, 0.151)	0.2770		
Pretreatment body mass index				
> 20.75 vs < 20.75	− 0.103 (− 0.368, 0.162)	0.4376		
ECOG performance status				
1, 2 vs 0	− 0.090 (− 0.356, 0.177)	0.5040		
Use of immune check point inhibitor				
Yes vs No	0.172 (− 0.104, 0.448)	0.2168	0.172 (− 0.104, 0.448)	0.2168
Gastrectomy				
Yes vs No	0.022 (− 0.246, 0.289)	0.8720		
Antiemetic Therapy				
Two-drug vs Three-drug	− 0.103 (− 0.368, 0.162)	0.4376		
Pretreatment serum albumin (g/dl)				
> 3.5 vs < 3.5	− 0.120 (− 0.388, 0.149)	0.3759		
Number of previous treatments				
3 vs 2	0.092 (− 0.194, 0.377)	0.5223		
Previous platinum regimen				
Yes vs No	− 0.146 (− 0.448, 0.156)	0.3368		
Previous smoking habit				
Yes vs No	− 0.011 (− 0.302, 0.280)	0.9393		
Drinking history within 30 days				
Yes vs No	− 0.042 (− 0.365, 0.280)	0.7942		

## Discussion

The EN-hance study evaluated antiemetic treatments for patients in Japan with unresectable and/or recurrent GC being treated with T-DXd via PROs of nausea and vomiting. The main finding was that neither the doublet regimen (palonosetron and dexamethasone) nor the triplet regimen (aprepitant/fosaprepitant, palonosetron, and dexamethasone) met the study-specified criteria for CR (no emetic events or rescue medication during 21 day study period) with T-DXd. Given that the primary endpoint was not met, and neither regimen was supportive, further research is needed to better characterize nausea and vomiting with T-DXd to tailor an anti-emetic regimen that suits the needs of the patients.

The reason why the present study did not meet the primary endpoints is as follows. First possible reason was that NK1 receptor antagonist (NK1RA) was not effective to prevent nausea and vomiting due to T-DXd pharmacokinetics. In the phase I study of T-DXd, the terminal elimination half-life of T-DXd (6.4 mg/kg, this dose was used in the present study) was 7.33 days [16]. Thus, pharmacokinetics of T-DXd might be reason of delayed CINV. On the other hand, the terminal elimination half-life of NK1RA which used in the present study reported as 11 h (orally) and 14 h (intravenous injection) [18]. Considering the differences of pharmacokinetics between T-DXd and NK1RA, NK1RA could not effectively prevent the CINV due to T-DXd. These explanations were supported by the antiemetic CR rate during the overall period was almost similar between the doublet regimen group and the triplet regimen group in the present study. Very recently, Sakai et al. evaluated the efficacy of an olanzapine-based triplet regimen for preventing nausea and vomiting in breast cancer patients receiving their first cycle T-DXd [19]. They reported that the delayed phase CR rate was significantly greater with olanzapine than placebo (70.0% versus 56.1%, *p* = 0.047). In Sakai’s study, taking into consideration the half-lives of T-DXd and olanzapine (33 h), they administered olanzapine 5 mg once daily for 6 days in their trial. The study setting, taking into account the pharmacokinetics of T-DXd and olanzapine, may have effectively suppressed CINV. Second possible reason is that the patients treated with triplet regimen seem to have disadvantageous backgrounds. When we analyzed the risk factors for nausea and vomiting in the present study, there was significant factor. The pretreatment BMI was a risk factor for vomiting. However, there was no significant difference between the two groups. On the other hand, there might be some not well-balanced factors such as experience of hyperemesis gravidarum and previous CINV in the present study. These factors might have negative impacts for the present study. Therefore, future study needs to concern the influence of experience of hyperemesis gravidarum, alcohol taking, and previous CINV on nausea and vomiting. The third possible reason was that the use of rescue antiemetic treatment was different. Although the protocol specified the type and use of rescue antiemetic treatment, there were some differences in the frequency and timing of rescue antiemetic treatment between two arms. According to Fig. 3A, B, in the double regimen, 6 patients received rescue antiemetic treatment within 3 days of onset of emetic events. In addition, among 6 patients, 4 patients continued rescue antiemetic treatment until emetic events disappeared. Six patients with double regimen who received rescue antiemetic treatment promptly and adequately did not repeat and continue the emetic events. On the other hand, in the triplet regimen, there was only one patient who received rescue antiemetic treatment within 3 days of onset of emetic events and continued rescue antiemetic treatment until emetic events disappeared. Considering these, in the management of emetic events associated with T-DXd treatment, one possibility is to use the rescue antiemetic treatment immediately after the appearance of emetic events and during emetic events. Therefore, to manage the emetic events associated with T-DXd treatment, the timing and duration of rescue antiemetic treatment might be important.

The safety analysis in a phase 1 study of T-DXd for GC showed that the incidence rate of vomiting was 52.5% in patients with no scheduled antiemetic treatment. Based on these reports, we set a threshold CR value of 45% in the present study. However, the CR rate was 37.9% in the present study. The discrepancy between CR in the clinical trial setting and in this analysis may have been due at least in part to different methods of evaluation. Physicians recorded the episodes of vomiting in the previous clinical trials, while patients recorded the episodes of vomiting in the present study. The discrepancy of adverse events recording between physicians and patients was well known [20]. Basch et al. compared symptomatic adverse events between PROs and investigator CTCAE evaluations [21]. They found that patients reported more adverse events than investigators for various symptoms. In particular, clinicians underreported anorexia, fatigue, nausea, and pain in comparison to patients. Thus, this study was set up with an incidence of emetic events based on the previous results reflecting physician judgment. Future studies aimed at optimizing prophylaxis of nausea and vomiting with T-DXd should consider the results of this study.

The present study showed that other AEs of any grade were observed in 51.7% of patients receiving the doublet regimen and 76.7% of patients receiving the triplet regimen, with grade 3 or 4 AEs occurring in at least 10.3% and 46.7% of patients, respectively. The rates of non-hematological AEs, such as fatigue (6.9% vs. 13.3%) and anorexia (17.2% vs. 26.7%), were similar. Conversely, hematological AEs, such as neutropenia (10.3% vs. 23.3%) and thrombocytopenia (3.4% vs. 16.7%), were more frequent in the triplet regimen group than in the doublet regimen group. However, according to previous reports, the incidence of each AE can be managed [11, 22].

Although our study did not meet the primary endpoint, T-DXd already is highly active in the clinic and getting used widely throughout so many patients with HER2-related cancer. Thus, special attention is required for our study results. Because our study included some limitations. First, this phase 2 study was an open-label design with a small number of patients, and each patient may be affected by a different risk of emesis. Second, since the predefined expected CR rates in this trial was based on physician-assessed results from previous trials, it may be difficult to compare PROs in the present study with other trials. However, our results were based on objective assessment of patient-reported outcome and showed that emetic events lasted longer than expected. Third, since this study evaluated only one cycle, it was not enough to assess the status of emetic events in subsequent treatment cycles or the impact on overall treatment. Therefore, future trials may require continued evaluation.

In conclusion, given that the primary endpoint was not met, further research is needed to better characterize nausea and vomiting with T-DXd to tailor an anti-emetic regimen that suits the needs of the patients.
